# A new species of *Sweltsa* Ricker, 1943 (Plecoptera, Chloroperlidae) and a supplementary description of *Sweltsahamula* Chen & Du, 2017 from China

**DOI:** 10.3897/BDJ.10.e86347

**Published:** 2022-07-07

**Authors:** Abdur Rehman, Qing-Bo Huo, Yu-Zhou Du

**Affiliations:** 1 School of Horticulture and Plant Protection & Institute of Applied Entomology, Yangzhou University, Yangzhou 225009, China School of Horticulture and Plant Protection & Institute of Applied Entomology, Yangzhou University Yangzhou 225009 China; 2 Joint International Research Laboratory of Agriculture and Agri-Product Safety, the Ministry of Education, Yangzhou University, Yangzhou 225009, China Joint International Research Laboratory of Agriculture and Agri-Product Safety, the Ministry of Education, Yangzhou University Yangzhou 225009 China

**Keywords:** Stoneflies, *Sweltsaliupanshana* sp. nov., *Sweltsahamula*, Ningxia, new species, China

## Abstract

**Background:**

The genus *Sweltsa* is a small to medium-sized stonefly with distinct coloured wings, giving the species the common name of green stoneflies. It belongs to the family Chloroperlidae. This genus includes more than 55 species world wide, 14 of which have been reported from China.

**New information:**

A new species of the genus *Sweltsa* Ricker, 1943, *Sweltsaliupanshana* Rehman, Du & Huo sp. nov. from Ningxia Hui Autonomous Region, Liupan Mountain, China is described; this is the second record of *Sweltsa* from Ningxia Hui Autonomous Region. In addition, the first female description and male supplementary description of *Sweltsahamula* Chen & Du, 2017 from Sichuan Province are provided. Diagnosis, description and colour illustration of the new species and of *Sweltsahamula* are provided and the morphological characteristics are compared with closely-related species.

## Introduction

The chloroperlid genus *Sweltsa* Ricker, 1943 belongs to the subfamily Chloroperlinae Okamoto, 1912. The genus *Sweltsa* was proposed by Ricker (1943) as a subgenus of *Alloperla* and was given generic status by Illies (1966). This genus is currently distributed in western and eastern Nearctic and eastern Palearctic/Oriental Regions (Stark and Baumann 2007, Kondratieff and Baumann 2009, Stark and Baumann 2021, DeWalt et al. 2022). Presently, 14 species of this genus are known from China (Wu 1938, Du 1999, Figueroa and Fochetti 2002, Stark and Sivec 2009, Li et al. 2014, Li et al. 2017, Chen and Du 2017, Yang and Li 2018, Dong et al. 2018, Mo et al. 2020, Li et al. 2021, Rehman et al. 2022a, Rehman et al. 2022b). Most of these recorded species bear a prominent transverse ridge on tergum 9 and this was previously considered as an obvious characteristic of the genus *Sweltsa*. Recently, Rehman et al. (2022b) proposed the *Sweltsarecurvata* group for those species that lack transverse ridge on tergum 9 and placed six Palearctic and two Nearctic species in that group. Amongst Chinese species, *S.recurvata* Wu, 1938, *S.wui* Stark & Sivec 2009, *S.ligula* Rehman, Huo & Du, 2022 and *S.lateoblonga* Rehman, Du & Huo, 2022 are added to the *Sweltsarecurvata* group. Previously, only *Sweltsalongistyla* Wu, 1938 was reported from Ningxia Hui Autonomous Region and Henan Province, possessing a prominent transverse ridge on tergum 9. Ningxia Hui is an autonomous region in northwest China, bordered by Shaanxi to the east, Gansu to the south and west and Inner Mongolia Autonomous Region to the north. In this study, we propose a new species of *Sweltsa* from Ningxia Hui Autonomous Region and Henan Province. The new species belongs to the *S.recurvata* group as defined by Rehman et al. (2022b), the male lacking the transverse ridge of tergum 9. Additionally, we provide the first female description and male supplementary re-description of *Sweltsahamula* Chen & Du, 2017 from Sichuan Province, which bears an obvious transverse ridge on tergum 9. Detail descriptions, illustrations and colour images of the new species and *Sweltsahamula* are provided and discussed.

## Materials and methods

The specimens were collected by aerial net or hand-picked and preserved in 75% ethanol. Terminalia were examined and illustrated by KEYENCE VHX-5000 and the final images were prepared using Adobe Photoshop CS6. The specimens were deposited in the Insect collection of Yangzhou University (ICYZU), Jiangsu Province, China. The morphological terminology of Surdick (1985), Stark and Sivec (2009), Li et al. (2021) and Rehman et al. (2022a) were followed.

## Taxon treatments

### Sweltsa liupanshana

Rehman, Du & Huo sp. n.

D53A1B09-ADA9-539D-BAA9-4500926A7C82

36C56C32-D1F6-48EA-A571-36000CDA1C81

Sweltsa Ricker, 1943
Type species: *Sweltsaoregonensis* Frison, 1935

#### Materials

- **Type status:** Holotype. **Occurrence:** recordedBy: Wang Zhi-Jie & Xue Hia Yang; individualCount: 1; sex: male; lifeStage: adult; occurrenceStatus: present; **Taxon:** kingdom: Animalia; phylum: Arthropoda; class: Insecta; order: Plecoptera; family: Chloroperlidae; genus: Sweltsa; specificEpithet: *liupanshana*; taxonRank: species; **Location:** continent: Asia; country: China; countryCode: CN; stateProvince: Ningxia; locality: Ningxia Hui Autonomous Region, Liupanshan (Liupan Mountain); minimumElevationInMeters: 2250; **Identification:** identifiedBy: Abdur Rehman, Du Yu-Zhou, Huo Qing-Bo; dateIdentified: 24-04-2022; **Event:** year: 2008; month: 8; day: 18; **Record Level:** language: en; institutionCode: ICYZU; basisOfRecord: PreservedSpecimen.
- **Type status:** Paratype. **Occurrence:** recordedBy: Wang Zhi-Jie & Xue Hia Yang; individualCount: 2; sex: female; lifeStage: adult; occurrenceStatus: present; **Taxon:** kingdom: Animalia; phylum: Arthropoda; class: Insecta; order: Plecoptera; family: Chloroperlidae; genus: Sweltsa; specificEpithet: *liupanshana*; taxonRank: species; **Location:** continent: Asia; country: China; countryCode: CN; stateProvince: Ningxia; locality: Ningxia Hui Autonomous Region, Liupanshan (Liupan mountain); minimumElevationInMeters: 2250; **Identification:** identifiedBy: Abdur Rehman, Du Yu-Zhou, Huo Qing-Bo; dateIdentified: 24-04-2022; **Event:** year: 2008; month: 8; day: 18; **Record Level:** language: en; institutionCode: ICYZU; basisOfRecord: PreservedSpecimen.
- **Type status:** Paratype. **Occurrence:** recordedBy: Du Yu-Zhou; individualCount: 2; sex: 1 male, 1 female; lifeStage: Adult; occurrenceStatus: present; **Taxon:** kingdom: Animalia; phylum: Arthropoda; class: Insecta; order: Plecoptera; family: Chloroperlidae; genus: Sweltsa; specificEpithet: *liupanshana*; taxonRank: species; **Location:** continent: Asia; country: China; countryCode: CN; stateProvince: Henan; locality: Baiyunnshan; minimumElevationInMeters: 1445; **Identification:** identifiedBy: Abdur Rehman, Du Yu-Zhou, Huo Qing-Bo; dateIdentified: 24-04-2022; **Event:** year: 1999; month: 7; day: 20; **Record Level:** language: en; institutionCode: ICYZU; basisOfRecord: PreservedSpecimen.

#### Description

**Figure 1.**
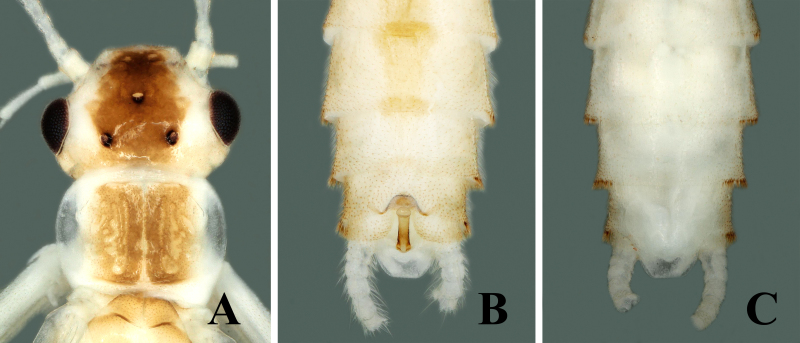
*Sweltsaliupanshana* Rehman, Du & Huo sp. n. Male **A** head and pronotum; **B** terminalia dorsal view; **C** terminalia ventral view.

Adult habitus: Triocellate. General body colour pale, yellow to brown in alcohol. Head with large, quadrate, median dark brown marking covering frons and ocellar areas; compound eyes black, ocelli brownish with black rings, median ocellus paler; antennae and palpi light brown (Fig. 1A). Pronotum disc with brown median portion narrowly surrounded by elongate paler rugosities; sides paler (Fig. 1A). Mesonotum and metanotum have dark brown U-shaped markings (Fig. 3A). Wings membrane transparent, legs pale. The anterior margin of abdominal tergum 1 brown, terga 2–7 have wide rectangular brown median stripe, tergum 8 has a small elliptical stripe anteromedially, lateral dark patches also being present on segments 1–4. Cerci pale brown, generally paler and covered with long setae (Fig. 3A).

##### Male

**Figure 2.**
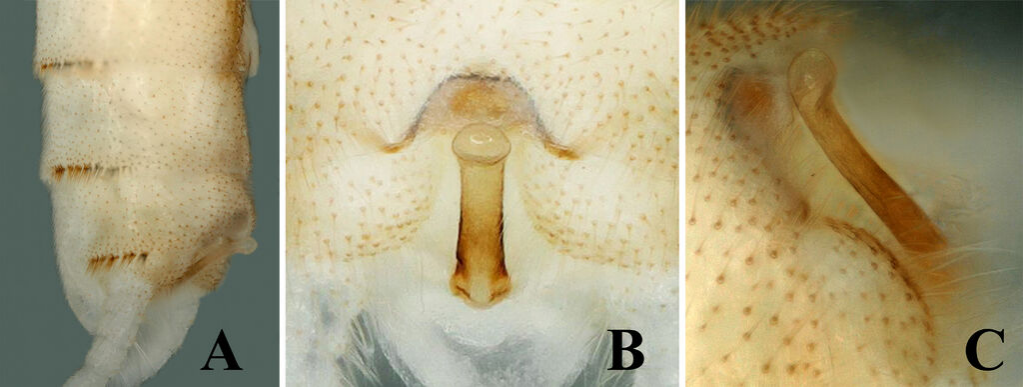
*Sweltsaliupanshana* Rehman, Du & Huo sp. n. Male **A** terminalia lateral view; **B** epiproct dorsal view; **C** epiproct lateral view.

Body length 6.5–7.0 mm, forewing length 6.0–6.5 mm, hind-wing length 5.0–5.5 mm (n = 2). Tergum 9 paler, without any transverse process or modification (Fig. 1B). Tergum 10 with sclerotised dark brown shield in middle (Fig. 1B and Fig. 2B). Sternum 9 ventrally with large trapezoidal subgenital plate, posterior margin truncate and extended over sternum 10 (Fig. 1C). Epiproct short and parallel from base to apex dorsally; apex swollen, shiny and rounded dorsally; lateral margins of epiproct from base to middle portion darker, medially brown (Figs 1, 2); laterally, epiproct dark brown, broad at base then slightly tapered apically, apex swollen and slightly bent upwards (Fig. 2A–C).

##### Female

**Figure 3.**
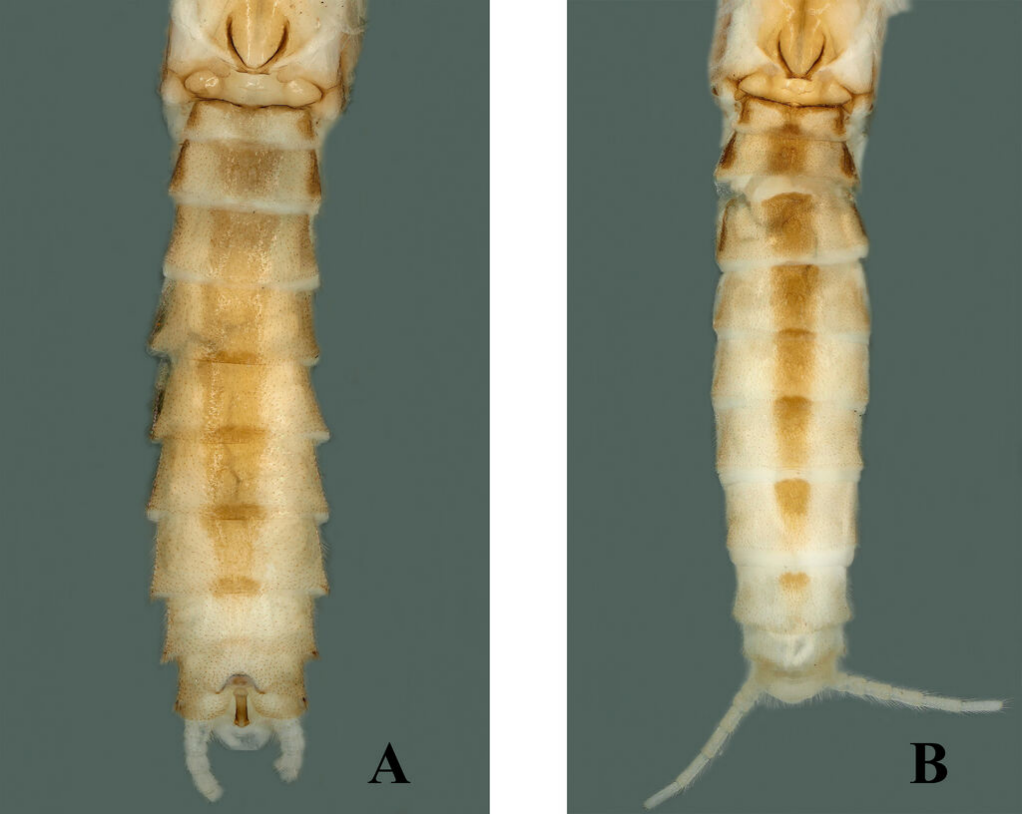
*Sweltsaliupanshana* Rehman, Du & Huo sp. n. **A** male abdomen, dorsal view; **B** female abdomen, dorsal view.

**Figure 4.**
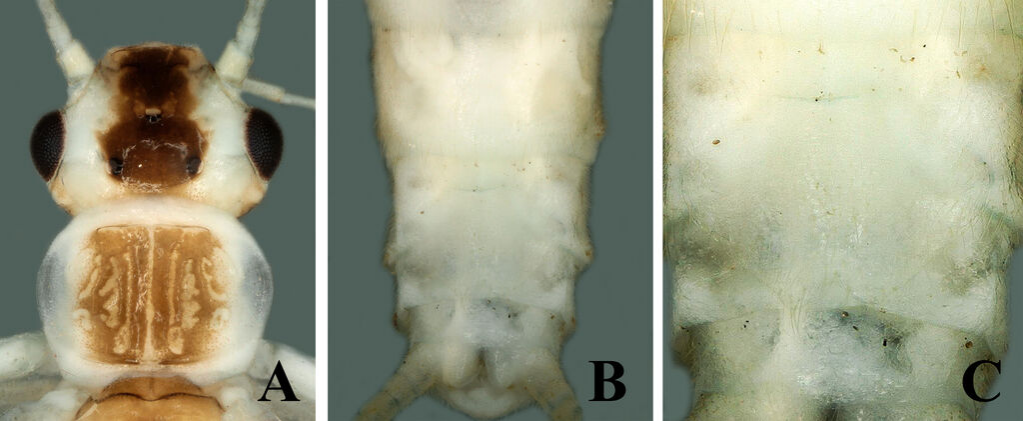
*Sweltsaliupanshana* Rehman, Du & Huo sp. n. Female **A** head and pronotum; **B** terminalia ventral view; **C** subgenital plate.

Body length 7.0–7.5 mm, forewing length 6.5–7.0 mm, hind-wing length 5.5–6.0 mm (n = 3). Habitus generally similar to male (Fig. 4A). Abdominal terga 1–6 dorsally with rectangular stripes, tergum 7 with oval shape and tergum 8 with small rounded stripe anteromedially (Fig. 3B). Posterior margins of sternum 8 bear large trapezoidal subgenital plate, covered with small hairs, reaching near posterior margins of sternum 9 and possess small rounded posteromedial notch. The sides of posterior notch covered with long fine hairs (Fig. 4B–C).

#### Diagnosis

The shape of the epiproct is characteristic of this new species. Epiproct short and almost parallel from base to apex dorsally, lateromedially sclerotised; apex swollen and rounded in dorsal view (Fig. 2B); in lateral view, epiproct parallel for its most part, except for swollen apex which is bent upwards (Fig. 2C).

#### Etymology

The species is named after the type locality, Liupan Mountain, also known by the Chinese name Liupanshan.

#### Distribution

China (Ningxia Hui Autonomous Region and Henan Province).

#### Taxon discussion

The new species pigmentation is mostly similar to *Sweltsabicurvata* (Li et al. 2021) and *Sweltsabrevihamula* (Dong et al. 2018), but these species possess a distinct sclerotised transverse ridge on tergum 9, while the new species lack that prominent morphological character. *Sweltsawui* Stark & Sivec, 2009, *S.recurvata* Wu, 1938 and *S.ligula* Rehman, Huo & Du, 2022 lack the tergum 9 transverse ridge similar to the new species and share some similar morphological characteristics with the new species. The head of *S.wui* bears a distinct triangular spot and the pronotum presents a dark median band (fig. 1 in Stark and Sivec 2009). In contrast, the head of the new species has a large rectangular median dark brown marking covering frons and ocellar areas (Fig. 1). The epiproct apex of *S.wui* bears a sclerotised cap, laterally much-expanded apically (figs. 6 and 7 in Stark and Sivec 2009), while the new species epiproct apex is swollen and lacks the cap and the lateral apex is slightly swollen upwards (Fig. 2A–C). Epiproct of *S.recurvata* is elongate, dorsally and laterally almost parallel from base to apex, apex pointed and, dorsolaterally, the tip is pencil-shaped (figs. 187–188 in Wu 1938), whereas the epiproct of the new species is much shorter, the apex is swollen like a bottle-cap and bent upwards in lateral view, easily differentiating the new species. The new species also show similar characteristics to *S.ligula* Rehman, Huo & Du, 2022. The epiproct of *S.ligula* is much larger and bears a spoon-shaped apex (figs. 1 and 3 in Rehman et al. 2022a), while the new species epiproct is short with a swollen apex. The female subgenital plate of the new species is similar to the *S.ligula* female, but can be easily distinguished by the body structure and pigmentation. The new species posterior notch of the subgenital plate is broader and sides of the notch possess fine hairs (Fig. 4C), while the *S.ligula* plate is sclerotised and the posterior notch lacks hairs which easily distinguish these two females.

### Sweltsa hamula

Chen & Du, 2017

213FC53F-8F48-5D67-9B39-DC003584FC1E

#### Materials

- **Type status:** Other material. **Occurrence:** recordedBy: Zhi-Teng Chen, Yue Shen; individualCount: 4; sex: 1 male, 3 female; lifeStage: adult; occurrenceStatus: present; **Taxon:** namePublishedInID: https://doi.org/10.11646/zootaxa.4337.2.8; scientificName: *Sweltsahamula* Chen & Du, 2017; acceptedNameUsage: *Sweltsahamula*; kingdom: Animalia; phylum: Arthropoda; class: Insecta; order: Plecoptera; family: Chloroperlidae; genus: Sweltsa; taxonRank: species; taxonomicStatus: accepted; **Location:** continent: Asia; country: China; countryCode: CN; stateProvince: Sichuan; locality: Mianyang City, Pingwu County, Wanglang National Nature Reserve, Baixionggou River; minimumElevationInMeters: 2886; verbatimLatitude: 33.0068; verbatimLongitude: 104.027; **Identification:** identifiedBy: Abdur Rehman & Du Yu-Zhou; dateIdentified: 24-04-2022; **Event:** year: 2017; month: 6; day: 25; **Record Level:** language: en; institutionCode: ICYZU; basisOfRecord: PreservedSpecimen.
- **Type status:** Other material. **Occurrence:** recordedBy: Zhi-Teng Chen, Yue Shen; individualCount: 3; sex: 2 male, 1 female; lifeStage: adult; occurrenceStatus: present; **Taxon:** namePublishedInID: https://doi.org/10.11646/zootaxa.4337.2.8; scientificName: *Sweltsahamula* Chen & Du, 2017; acceptedNameUsage: *Sweltsahamula*; kingdom: Animalia; phylum: Arthropoda; class: Insecta; order: Plecoptera; family: Chloroperlidae; genus: Sweltsa; taxonRank: species; taxonomicStatus: accepted; **Location:** continent: Asia; country: China; countryCode: CN; stateProvince: Sichuan; locality: Mianyang City, Pingwu County, Wanglang National Nature Reserve, Muyangchang; minimumElevationInMeters: 2589; verbatimLatitude: 32.9699; verbatimLongitude: 104.1029; **Identification:** identifiedBy: Abdur Rehman & Du Yu-Zhou; dateIdentified: 24-04-2022; **Event:** year: 2017; month: 6; day: 22; **Record Level:** language: en; institutionCode: ICYZU; basisOfRecord: PreservedSpecimen.

#### Description

##### Supplementary description

**Figure 5.**
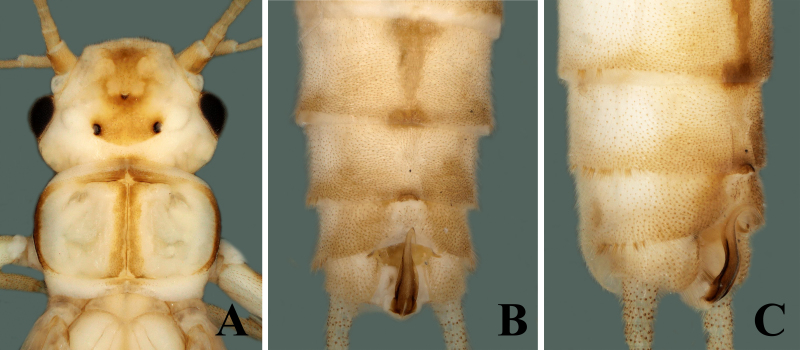
*Sweltsahamula* Chen & Du, 2017. Male **A** head and pronotum; **B** terminalia dorsal view; **C** terminalia dorsolateral view.

**Figure 6.**
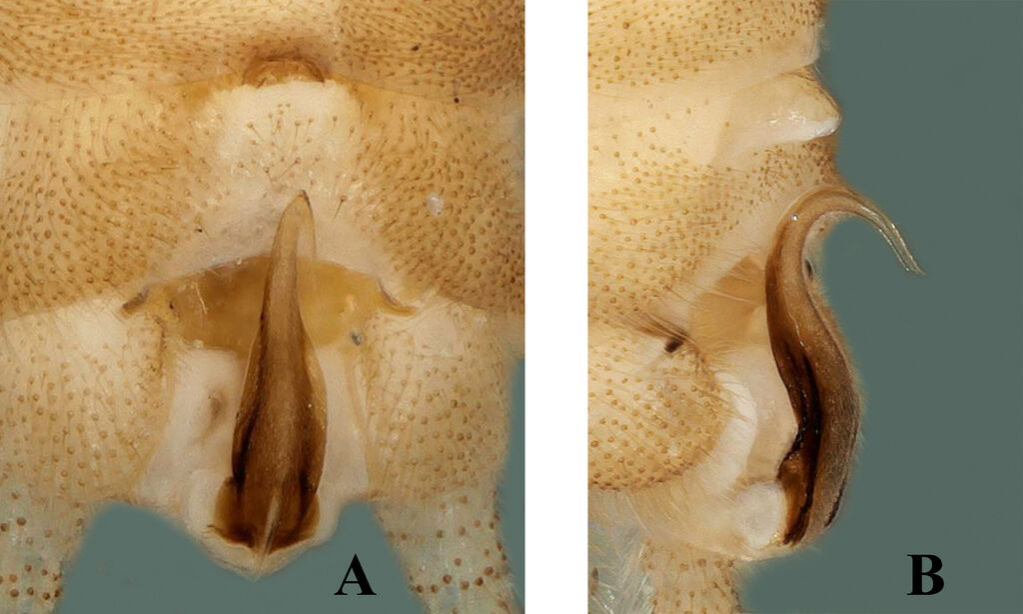
*Sweltsahamula* Chen & Du, 2017. Male **A** epiproct dorsal view; **B** epiproct lateral view.

Male head with median dark brown marking covering frons and ocellar areas. Pronotum disc with median brown band, medially pale with light rugosities, margins dark brown (Fig. 5A). Mesonotum and metanotum with dark U-shaped marking. Tergum 9 anteriorly with prominent transverse ridge (Fig. 5B). Tergum 10 divided medially, forming sclerotised shield-like structure. Epiproct wide and elliptical, medially slightly upraised, sides with upward dark margins from base to basal half dorsally; then gradually tapering to sharp apex, apically narrow and thread-like, bending backwards forming hook-shaped structure (Fig. 5B and Fig. 6A). Laterally sclerotised, constricted from base to basal half, then curved outwards, apically back-curved and forming hook-shaped apex (Fig. 5C and Fig. 6B).

##### Female

**Figure 7.**
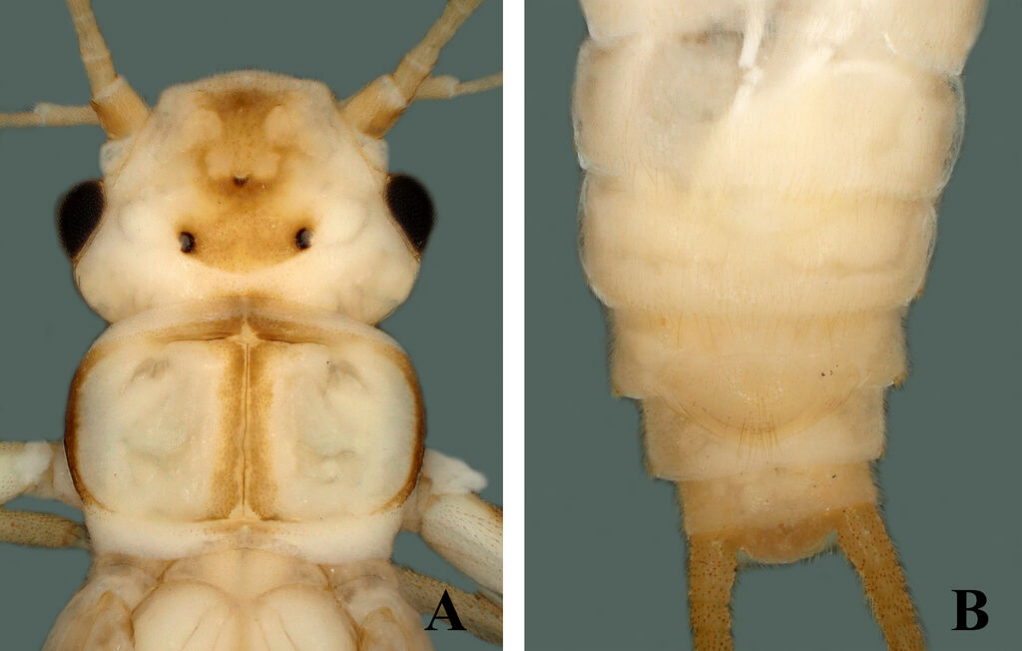
*Sweltsahamula* Chen & Du, 2017. Female **A** head and pronotum; **B** terminalia ventral view.

Body length 9.0–9.5 mm, forewing length 8.0–8.5 mm, hind-wing length 7.0–8.0 mm (n = 4). Habitus generally similar to male (Fig. 7). Abdominal tergum stripes same as male, except for tergum 8 with median brown elliptical mark; lateral dark patches present anteriorly on segments 2–4. Posterior margin of sternum 8 bears large rounded subgenital plate ventrally, reaching near to half of sternum 9. Subgenital plate posteromedially bears lobe-like structure, seems like U-shaped narrow patch and covered with many hairs (Fig. 7B).

#### Distribution

China, Sichuan Province, Wanglang National Nature Reserve.

#### Taxon discussion

This species was established by Chen and Du (2017). They did not provide any closely-related species maybe because this species is easily distinguishable from all *Sweltsa* by the epiproct backward hook. However, here we can add that this species epiproct structure is partially similar to *Sweltsailliesi* Zhiltzova & Levanidova, 1978 (Teslenko and Zhiltzova 2009) in general appearance and can be easily distinguished from this species by the epiproct backward hook. Besides this, we also describe the female of this species, which is not reported in the original description. The female subgenital plate of the *S.hamula* species is wide, rounded and posteromedially bears a small U-shaped patch and is covered with hairs (Fig. 7B). In contrast, the subgenital plate of *S.illiesi* is subtriangular and medially with a round patch and margin of the plate is covered with hairs (fig. 520 in Teslenko & Zhiltzova 2009). These features easily distinguish female of this species.

## Supplementary Material

XML Treatment for Sweltsa liupanshana

XML Treatment for Sweltsa hamula
